# Impact of Collection Method on Assessment of Semen HIV RNA Viral Load

**DOI:** 10.1371/journal.pone.0023654

**Published:** 2011-08-19

**Authors:** Brendan J. W. Osborne, Prameet M. Sheth, Colin Kovacs, Tony Mazzulli, Rupert Kaul

**Affiliations:** 1 Department of Medicine, University of Toronto, Toronto, Canada; 2 Department of Immunology, University of Toronto, Toronto, Canada; 3 Maple Leaf Medical Centre, Toronto, Canada; 4 Department of Medical Microbiology, Mount Sinai Hospital, Toronto, Canada; University of California San Francisco, United States of America

## Abstract

**Background:**

The blood HIV RNA viral load is the best-defined predictor of HIV transmission, in part due to ease of measurement and the correlation of blood and genital tract (semen or cervico-vaginal) viral load, although recent studies found semen HIV RNA concentration to be a stronger predictor of HIV transmission. There is currently no standardized method for semen collection when measuring HIV RNA concentration. Therefore, we compared two collection techniques in order to study of the impact of antiretroviral therapy on the semen viral load.

**Methodology/Principal Findings:**

Semen was collected by masturbation from HIV-infected, therapy-naïve men who have sex with men (MSM) either undiluted (Visit 1) or directly into transport medium (Visit 2). Seminal plasma was then isolated, and the HIV RNA concentration obtained with each collection technique was measured and corrected for dilution if necessary. Collection of semen directly into transport medium resulted in a median HIV RNA viral load that was 0.4 log10 higher than undiluted samples.

**Conclusions/Significance:**

The method of semen collection is an important consideration when quantifying the HIV RNA viral load in this compartment.

## Introduction

Globally there were an estimated 2.6 million new HIV-1 (HIV) infections in 2009 [1], most acquired through sex. The blood HIV RNA viral load is the best defined predictor of HIV transmission [2], probably because it is easily measured and tends to correlate with the genital tract (semen or cervico-vaginal) viral load [3]. However, recent studies have found that the semen HIV RNA viral load is a stronger independent predictor of HIV transmission than the blood viral load [4].

Following the initiation of antiretroviral therapy (ART) blood HIV RNA concentrations generally decrease to undetectable levels, in association with a 92% reduction in HIV transmission risk in a recent observational study [5]. However, a significant minority of individuals continue to have detectable levels of viral RNA in semen despite an undetectable HIV RNA blood VL, sometimes at very high levels [6]. Whether this phenomenon underpins the inability of ART to completely prevent HIV transmission is not clear. Research studies to clarify these issues will require well-validated assays to measure semen HIV RNA viral load, something which is more technically challenging than measurement of the blood VL due to the presence in semen of PCR inhibitors, endonucleases and other factors [7]. While commercially available molecular assays may be more reliable and reproducible than in-house assays [8], in this study we evaluated the impact of different semen collection methods on the HIV RNA level in ART-naïve men.

## Methods

### Human Subjects

HIV-infected, antiretroviral therapy-naïve men who have sex with men (MSM) were recruited through the Canadian Immunodeficiency Research Collaborative at the Maple Leaf Medical Clinic in Toronto, Canada. Participants were excluded if at either visit they had clinical urethritis, genital ulcer disease, laboratory evidence of infection by *C. trachomatis*, or *N. gonorrhoeae* by urine nucleic acid amplification testing (NAAT: Amplicor CT/NG assay, Roche Diagnostic Systems), or active *T. pallidum* infection by serology (RPR; rapid plasma reagin). A first-void urine dipstick for leukocytes was also performed to screen for asymptomatic urethritis. All participants provided informed, written consent; ethical approval for this study was obtained through the research ethics board of the University of Toronto.

### Sample acquisition, processing and viral load measurement

Paired blood and semen specimens were collected within an hour of each other at two separate study visits. Semen samples were collected by masturbation into a dry sterile container (undiluted) at visit 1, and directly into 10 mL of sterile RPMI 1640 (Gibco) containing 100 U/mL penicillin and 100 mg/mL streptomycin (Gibco) (transport medium) at visit 2. All study participants agreed to abstain from sexual intercourse or masturbation for 48 hours prior to sample donation. All samples were processed within 2 hours of collection. Seminal plasma was cryopreserved at −80°C after sample centrifugation at 850 g for 10 minutes. Blood plasma was collected and cryopreserved after ficoll density gradient centrifugation at 500 g for 25 minutes. Blood and semen plasma HIV-1 RNA concentrations were measured in the Mount Sinai Hospital Department of Microbiology (accredited by the Ontario Public Health Lab for clinical HIV-1 viral load measurement) using the Versant HIV-1 RNA 3.0 assay (bDNA; Bayer Diagnostics; lower limit of detection, 50 RNA copies/mL). Correction for semen dilution at visit 2 was calculated based on the total sample volume provided; since transport medium was occasionally spilled during semen collection, where the returned total volume (semen and transport medium) was lower than the original volume of transport medium, we assumed a semen volume of 2 ml (the mean volume of undiluted samples collected during visit 1).

### Statistical analysis

All analyses were formed with SPSS software (version 18; SPSS). Data were statistically analyzed using the non-parametric paired Wilcoxon signed rank test for median measurements and changes in mean VL measurements. Statistical significance was defined as p<0.05.

## Results

Twenty-seven participants were recruited; the median CD4^+^ T cell count was 550/mm^3^ (range, 320–1210 mm^3^) at visit 1 and 470/mm^3^ (range, 160–780 mm^3^) at visit 2. There was no statistically significant difference in the CD4 counts between visits (Wilcoxon paired p = 0.383), although one individual at visit 2 had progressed to AIDS based on a CD4 count <200 mm^3^ (160 mm^3^). No participant had a prior history of an AIDS-defining illness (at either study visit), and no participant had syphilis, *N. gonorrhea* or *C. trachomatis* infection by NAAT, clinical urethritis, genital ulcer disease or leukocytes detected on dipstick of first void urine. Study visits were a median of 6 months apart, and there was no difference in the blood HIV RNA VL (4.26 vs. 4.35 log_10_ RNA copies/mL, Visit 1 vs. Visit 2; p = 0.274) between visits. Five participants (18.5%) had an undetectable semen VL by at least one of the two collection methods (i.e.: at ε1 study visit), and in 4/5 the semen VL was undetectable at both study visits: for practical reasons these 4 participants were not included in the comparison of sampling techniques.

**Figure 1:**
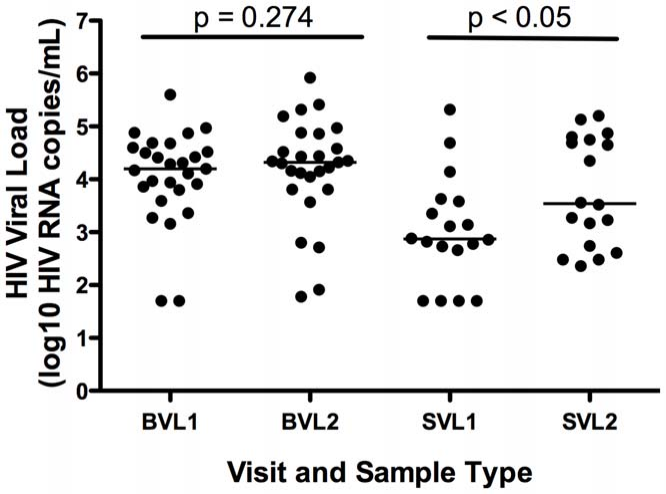
Blood and semen HIV RNA viral load. Blood was collected and the HIV RNA viral load assayed the same way at both study visits (BVL1 and BVL2, respectively); semen was collected undiluted at visit 1 (SVL1) and directly into transport medium and visit 2 (SVL2). Participants with an undetectable semen viral load at both study visits were excluded from statistical analysis.

In those participants with a detectable semen VL during at least one study visit, the median HIV load as measured in undiluted semen (Visit 1) was 0.42 log_10_ copies/mL (2,236 copies/mL) lower than that measured in semen that had been collected directly into transport medium. Median semen HIV RNA collected undiluted was 3.14 log_10_ copies/mL (1,396 RNA copies/mL, range, 50- 210,350 RNA copies/mL) vs. 3.56 log_10_ copies/mL (3,631 RNA copies/mL, range, <300–1,002,030 RNA copies/mL) when collected into transport media (Figure 1; p = 0.012).

The proportion of participants with any detectable HIV RNA in semen did not vary by collection technique (21/27 undiluted vs. 20/27 diluted; p = NS). As expected, having an undetectable semen VL at one visit increased the probability of remaining undetectable at the next (LR = 6.0; p = 0.014). An estimated semen volume of 2 mL was used at visit 2 for 14/27 participants who had spilled transport medium during sample collection (see Methods section, above). When these participants were excluded, our overall results were unchanged with a median semen HIV concentration of 2.88 log_10_ at visit 1 and 3.56 at visit 2 (median difference 0.68 log_10_, p = 0.033).

## Discussion

This study demonstrates that the method of semen collection can have a substantial impact on semen HIV RNA VL measurements, and this should be an important consideration when performing and assessing studies of semen HIV RNA load. The cause of the reduced level when measuring the HIV RNA VL in semen that had been collected undiluted is not clear, but might relate to PCR inhibitors present in undiluted semen [9] or to the presence in semen of various enzymes and other immune factors [10]–[11]; certainly, seminal plasma is well described to have substantial cytotoxic effects [12]. Interestingly, while the semen viral load was significantly higher when collected into transport medium, there was no difference between collection methods in the proportion of participants who had any detectable semen HIV RNA. We hypothesize that this may be because although collection into RPMI medium was associated with an increased semen HIV RNA level, this also diluted the sample approximately six-fold, decreasing our assay limit of detection from δ50 to δ300 HIV RNA copies/mL.

The second study visit, when semen VL was assayed in a diluted sample, was 6 months after the semen VL was measured undiluted. This raises the possibility that a higher semen VL might represent HIV disease progression. However, the fact that both the blood VL and CD4+ T cell count were unchanged between visits strongly suggests that this was not the case. The semen HIV concentration may be more variable than that in blood plasma, and in addition there is considerable variability of all HIV RNA assays currently in clinical use. However, neither of these sources of variability would explain our observation that the semen HIV viral load was significantly and consistently higher when samples were collected into transport medium rather than undiluted; indeed, such random variability would tend to have blunted our ability to find such a difference. In addition, in those participants with a spilled sample at visit two, if we assumed a volume equal to their first sample, the difference in semen viral loads across the two collection methods was still statistically significant.

Overall, our findings suggest that semen collection technique is an important consideration if the semen viral load is to be assessed quantitatively, since immediate collection of semen into transport medium was associated with higher semen HIV RNA concentration that that measured into semen collected undiluted. However, this would less critical if the goal were to deem semen HIV RNA as being “detectable” or “undetectable”, since these proportions were not altered by collection technique.
